# Instantaneous Respiratory Estimation from Thoracic Impedance by Empirical Mode Decomposition

**DOI:** 10.3390/s150716372

**Published:** 2015-07-07

**Authors:** Fu-Tai Wang, Hsiao-Lung Chan, Chun-Li Wang, Hung-Ming Jian, Sheng-Hsiung Lin

**Affiliations:** 1 Department of Electrical Engineering, Hwa Hsia University of Technology, 111, Gongzhuan Rd., Zhonghe, New Taipei City 23568, Taiwan; E-Mail: wft.intuitive@seed.net.tw; 2 Department of Electrical Engineering, Chang Gung University, 259 Wenhwa 1st Road, Kweishan, Taoyuan 33302, Taiwan; E-Mails: zxccc45697@gmail.com (H.-M.J.); aegis793@hotmail.com (S.-H.L.); 3 Department of Cardiology, Chang Gung Memorial Hospital, 5 Fu-Hsing Street, Kweishan, Taoyuan 33305, Taiwan; E-Mail: wang3015@cgmh.org.tw

**Keywords:** impedance plethysmography, respiratory disorder, empirical mode decomposition, intrinsic mode function

## Abstract

Impedance plethysmography provides a way to measure respiratory activity by sensing the change of thoracic impedance caused by inspiration and expiration. This measurement imposes little pressure on the body and uses the human body as the sensor, thereby reducing the need for adjustments as body position changes and making it suitable for long-term or ambulatory monitoring. The empirical mode decomposition (EMD) can decompose a signal into several intrinsic mode functions (IMFs) that disclose nonstationary components as well as stationary components and, similarly, capture respiratory episodes from thoracic impedance. However, upper-body movements usually produce motion artifacts that are not easily removed by digital filtering. Moreover, large motion artifacts disable the EMD to decompose respiratory components. In this paper, motion artifacts are detected and replaced by the data mirrored from the prior and the posterior before EMD processing. A novel intrinsic respiratory reconstruction index that considers both global and local properties of IMFs is proposed to define respiration-related IMFs for respiration reconstruction and instantaneous respiratory estimation. Based on the experiments performing a series of static and dynamic physical activates, our results showed the proposed method had higher cross correlations between respiratory frequencies estimated from thoracic impedance and those from oronasal airflow based on small window size compared to the Fourier transform-based method.

## 1. Introduction

Monitoring of respiratory activity is useful for detecting respiratory disorders, such as the sleep apnea, cessation of breathing in infants, shortness of breath in patients with heart failure, and so on. Respiratory sensor belts or clothes based on inductive plethysmography, strain gauges, or piezoelectric sensors can measure the changes of thoracic and abdominal volumes that are caused by inspiration and expiration. This kind of measurement is more convenient and comfortable than gas flow measurement through a mouthpiece or a mask. However, the respiratory sensor belt should have a close attachment so that the expansion and distraction of thoracic and abdominal walls can be detected by the sensor. The sensor belt usually needs adjustment to maintain close attachment when posture or lying position is changed, whereas it is cumbersome for ambulatory recording or long-term monitoring.

Impedance plethysmography provides an alternative for measuring respiratory activity by injecting a small alternative current into the body, detecting the pass-through voltage across electrodes, then computing the body impedance. As the thoracic wall expands during inspiration, the thoracic impedance increases, and decreases during exhalation. The impedance-based measurement has an advantage in that the sensor is just the human body itself. It is not necessary to adjust the electrodes as body position changes as long as the electrodes adhere to the body. Since there is no belt surrounding, impedance plethysmography causes less pressure on the body than a respiratory sensor belt. Therefore, impedance-based measurement is suitable for ambulatory monitoring of respiratory activity.

**Figure 1:**
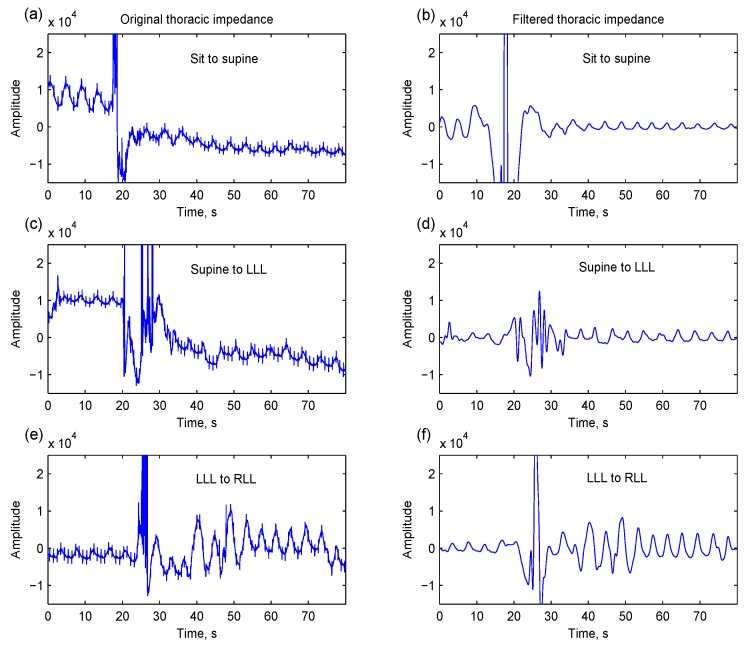
Three thoracic impedance segments contain motion artifacts caused by various postural changes: (**a**) sit to supine; (**c**) supine to left-lateral lying (LLL); and (**e**) LLL to right-lateral lying (RLL). These movement-induced artifacts cannot be easily removed by digital filtering (a fourth-order anti-causal Butterworth highpass filter with a cutoff frequency of 0.1 Hz and a sixth-order anti-causal Butterworth lowpass filter with a cutoff frequency of 1 Hz). The respirations are still overwhelmed by motion artifacts, which may be regarded as respirations (**b**,**d**,**f**).

During ambulatory recording, sudden or large upper-body movement usually disturbs the sensing of thoracic expansion and distraction. As shown in Figure 1, the measured thoracic impedance is overwhelmed by large artifacts. The movement-induced artifact cannot be easily removed by digital filtering; moreover, the filtering creates a pseudo-respiration problem.

Normal respiration is characterized by a regular, wavy pattern that reflects the tidal change of pulmonary volume. However, the respiration signal is not always stationary. The amplitude or frequency of breathing may change over time. In particular, respiratory disorders usually have specific respiratory episodes such as obstructive sleep apnea, Cheyne–Stokes respiration, and so on. The number of obstructive sleep apnea episodes per hour provides an index for case identification and severity assessment [1]. The percentage of nocturnal Cheyne–Stokes respiration is demonstrated as a predictor of mortality or prognosis in patients with chronic heart failure [2,3].

Empirical mode decomposition (EMD) is a new method for analyzing a non-stationary signal. Numbers of intrinsic mode functions (IMFs) are extracted directly from the signal [4]. The local properties of non-stationary signal can be captured by the IMFs. Recently, several studies have demonstrated the advantage of EMD in capturing time-related features of biomedical signals. The time-dependent median frequency derived from the IMFs of electromyography of quadriceps muscles gives a more reliable assessment of muscle fatigue during cyclic dynamic contraction than the Fourier- and wavelet-based methods [5]. The time-amplitude index derived from the fluctuated time series of pain-relief demands from patient-controlled analgesia through EMD is significantly related to the visual analog scale, a measure of pain intensity by interviewers in postoperative patients [6]. EMD is also used to characterize temporal features of slow- and fast-wave oscillations in an electroencephalogram for estimating the depth of sleep and discriminating rapid eye movement sleep [7]. Moreover, EMD is demonstrated to be efficient in noise or artifact reduction. Electromyogram noises, power line interferences, and baseline wanders can be removed from the electrocardiograms with minimum signal distortion [8,9] for feature enhancement [10] and QRS detection [11]. Cerebral activities in the ocular-related components derived from electroencephalograms can be removed by the EMD in order to cancel ocular artifacts in the electroencephalograms [12]. Furthermore, ventricular fibrillation can be extracted from the corrupted electrocardiogram due to cardiopulmonary resuscitation-related fluctuation by the EMD [13]. Tissue artifacts are more efficiently removed by the EMD than by lowpass filtering based on the simulated noisy respirations and real signals measured from piezoelectric sensor belts during walking and running [14].

**Figure 2:**
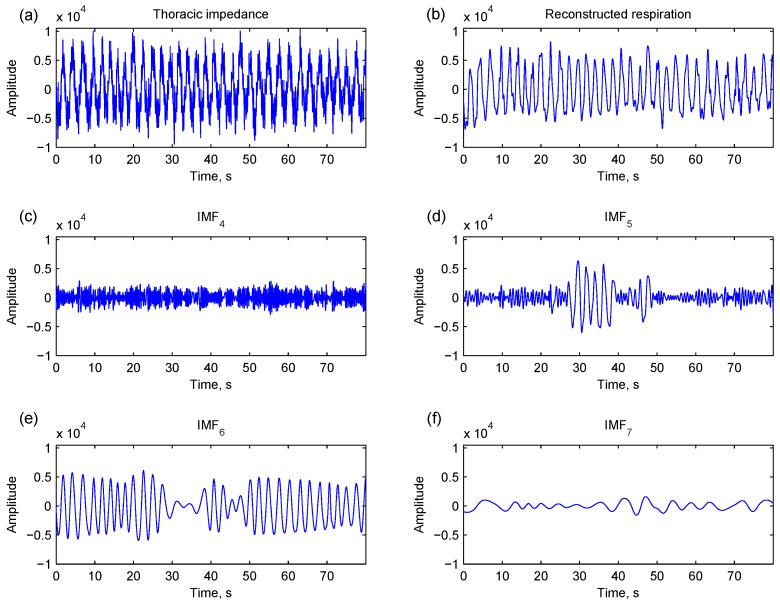
A thoracic impedance signal (**a**) is decomposed into numbers of intrinsic mode components (IMFs) by empirical mode decomposition (**c**–**f**) for respiration reconstruction (**b**). Major respiratory components are present at local points of IMF_5_ (**d**) and IMF_6_ (**e**).

The decomposed IMFs have well-behaved Hilbert transforms so that instantaneous frequencies and amplitudes can be derived from the IMFs. The instantaneous estimation is beneficial for capturing respiratory episodes from normal respirations. However, only some of the IMFs are related to respiration, so identifying respiration-related IMFs is needed for estimating instantaneous properties. Liu *et al.* applied mutual information ratio and power of IMFs to select the best IMFs to reconstruct respiration signals [14]. However, even if respiratory components are similar, they may be decomposed at different local points in adjacent IMFs. As shown in Figure 2, a major respiratory component is present at both IMF_5_ and IMF_6_. Therefore, the local properties as well as global properties of IMFs should be considered for identifying respiration-related IMFs.

In this paper, a strategy to detect induced motion artifacts in the thoracic impedance is proposed to avoid incorrectly regarding motion artifacts as respiratory components. EMD analysis is used to decompose the thoracic impedance into several IMFs. Respiration-related IMFs are identified using a novel rule based on both global and local properties of IMFs. Instantaneous respiratory properties are therefore estimated from the respiration-related IMFs. The capability of EMD analysis in estimating respiratory frequency was validated against the experiments during static postures and dynamic physical activities with parallel recording of oronasal airflow.

## 2. Methods

### 2.1. Respiration Measurements and Data Preprocessing

Ten healthy male subjects (24.5 ± 1.4 y/o, 167.3 ± 5.2 cm, 65.8 ± 14.4 kg) performed a series of physical activities including supine, left-lateral lying, right-lateral lying, sitting, standing, slow walking (90 cycles per minute, cpm), fast walking (120 cpm), slow running (140 cpm), fast running (160 cpm), and then recovering from standing to supine. Each physical activity lasted for 3 min. Three-lead electrocardiogram (leads I, II and precordial) and thoracic impedance (ADS1294R, Texas Instruments, TX, USA) were recorded with a sampling rate of 250 Hz in a portable device (Kangyi Electronics, Taiwan). Meanwhile, oronasal airflow was parallel digitized into the portable device through a mask connected to a pneumotach airflow transducer and a transducer amplifier (TSD107B and DAC100C, Biopac Systems, Goleta, CA, USA). The protocol of this study was approved by the local Research Ethics Committee. The participants gave their informed consent.

The thoracic impedance was filtered by a fourth-order anti-causal Butterworth highpass filter with a cutoff frequency of 0.1 Hz to suppress low-frequency baseline wandering. The oronasal airflow was also filtered by a sixth-order anti-causal Butterworth lowpass filter with a cutoff frequency of 1 Hz to suppress high-frequency noises.

### 2.2. EMD-Based Respiration Analysis

**Figure 3:**
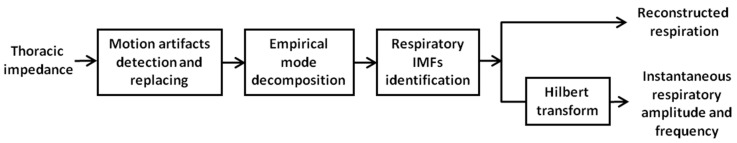
A block diagram of the proposed respiration analysis. Motion artifacts are first detected and replaced by mirrored data from the prior and the posterior. Numbers of intrinsic mode functions (IMFs) are subsequently computed through empirical mode decomposition. Respiration-related IMFs are identified and used to reconstruct respiration and compute instantaneous frequency and amplitude.

Figure 3 depicts the block diagram of EMD-based respiration analysis. First, an artifact detection and replacing algorithm is applied to detect the artifacts induced by postural changes or other motion disturbances. The affected portion is replaced by the mirror data from the prior and posterior. Then, EMD is applied to decompose this signal into numbers of IMFs. Respiration-related IMFs are identified according to an intrinsic respiratory reconstruction index. The identified IMFs are used to reconstruct respiration and estimate the instantaneous respiratory frequency and amplitude.

#### 2.2.1. Artifact Detection and Replacing

**Figure 4:**
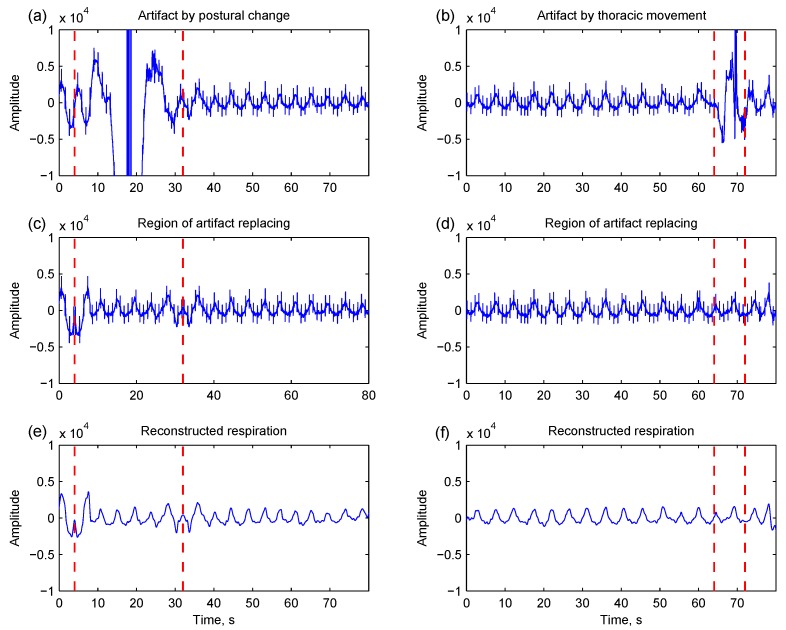
Two thoracic impedance segments are corrupted by motion artifacts caused by postural change (**a**) and thoracic movement (**b**), respectively. Through the artifact detection and replacing, the region of the artifact is identified (highlighted by dashed lines), and the affected portion is replaced by the mirror data from the prior and posterior (**c**,**d**); The respirations are therefore reconstructed by the empirical mode decomposition-based method (**e**,**f**).

Respiration-irrelevant thoracic movement will disturb the measured body impedance based on the change of thoracic volume. The scale and type of motion determine the disturbance’s intensity. Figure 4a shows an example of motion artifact caused by postural change. Figure 4b shows another example of motion disturbance induced by thoracic movement. Because EMD is a data-driven and self-adaptive method, large artifacts will interfere with EMD processing so that the respiration-related component cannot be distinctly captured by the IMFs. To overcome this difficulty, an artifact detection and replacing algorithm is proposed.

A bin-sorting method [15] is used to determine a threshold for detecting motion artifacts. The thoracic impedance is first divided into consecutive 8 s bins with 50% overlapping. The signal intensities, defined as the standard deviation of data, of all bins are sorted in ascending order. The intensities below 50% are averaged and 10 multiples of the average are set to the detection threshold. The respiration signal is re-divided into consecutive 4 s bins. The bins whose intensities are greater than the threshold are marked as possible artifact bins. The nearby marked bins with a time interval <4 s are regarded as from the same artifact. Thereby the region of artifact is defined. The data within the region are removed. The former half are replaced by the replicas mirrored from the data prior to the range; the latter half were mirrored from the posterior.

#### 2.2.2. Empirical Mode Decomposition

EMD is a data-driven analysis method in that the analyzed signal does not need to be stationary and linear. Compared to the basis-based methods such as the Fourier transform, Wavelet transform, *etc.*, several intrinsic mode functions (IMFs) are extracted directly from the analyzed signal *x*(*t*) through EMD [4]. Each IMF decomposition starts from *h*_0_(*t*), equal to *x*(*t*) for the first decomposition. Two envelopes are constructed by the local minima and local maxima of *h*_0_(*t*) separately. A differential signal *h*_1_(*t*) is obtained by subtracting *h*_0_(*t*) from the mean of these two envelopes. The differential signal is an IMF if two conditions are met: the number of zero crossings and the number of extrema are either equal or differ by one; and the mean value of the two envelopes is zero at any point. This process is named as the sifting process. The differential signal is ideally an IMF. If it is not, the sifting process repeats until the following criterion is satisfied [16]:

(1) $$0.2<\sum_{t=0}^{T}\left[\frac{{\left|h_{k-1}(t)-h_{k}(t)\right|}^{2}}{h_{k}^{2}(t)}\right]<0.3$$

The IMF is therefore set to *h_k_*(*t*).

The derived IMF represents an oscillation mode embedded in the signal *x*(*t*). It can be either a narrow band signal or non-stationary component. The above process continues based on the residual data defined as

(2) $$r_{1}=x\text{(}t\text{)}-{\text{IMF}}_{1}$$

which may contain another component with a longer period. The second IMF and the second residual signal are therefore derived. The decomposition repeats until the IMF conditions are no longer satisfied:

(3) $$\begin{array}{l} r_{2}=r_{1}-{\text{IMF}}_{2}\\ \;\vdots \\ r_{n}=r_{n-1}-{\text{IMF}}_{n} \end{array}$$

The input signal is therefore expressed by

(4) $$x\text{(}t\text{)}=\sum_{j=1}^{n}{\text{IMF}}_{j}+r_{n}$$

where *n* is the number of IMFs.

#### 2.2.3. Identifying Respiration-Related IMFs

**Figure 5:**
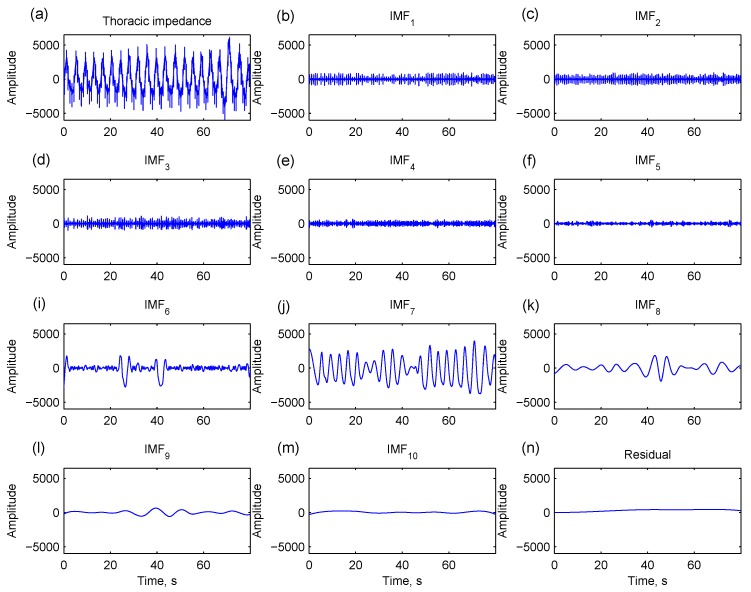
A thoracic impedance signal (**a**) is decomposed into 10 intrinsic mode functions (IMFs) and a residual signal by empirical mode decomposition. IMF_1_…IMF_5_ have too many zero-crossing points, containing noises rather than respiratory patterns (**b**–**f**). IMF_6_ and IMF_7_ contain major respiratory component (**i**,**j**). IMF_8_…IMF_10_ (**k**–**m**) and residual signal (**n**) are low-frequency structures of the respiration.

Through EMD, the thoracic impedance is decomposed into several IMFs of different oscillatory modes. Zero-crossing is one of the intrinsic properties for each IMF. An IMF with more zero-crossing points contains oscillatory components with higher frequencies and *vice versa*. As illustrated in Figure 5, IMFs with too many zero-crossing points contain noises rather than respiratory patterns (IMF_1_…IMF_5_). An intrinsic respiratory reconstruction index (IRRI) is defined to exclude IMFs with too many zero-crossing points (IMF*_j_*, *j* < IRRI) and select the rest of the IMFs for respiration reconstruction (*j* ≥ IRRI). IMFs that contain fewer zero-crossing points (IMF_8_…IMF_10_ in Figure 5) and a residual signal are included because they are low-frequency structures of a respiration. The respiration is therefore reconstructed by

(5) $$x\text{(}t\text{)}=\sum_{j=IRRI}^{n}{\text{IMF}}_{j}+r_{n}$$

**Figure 6:**
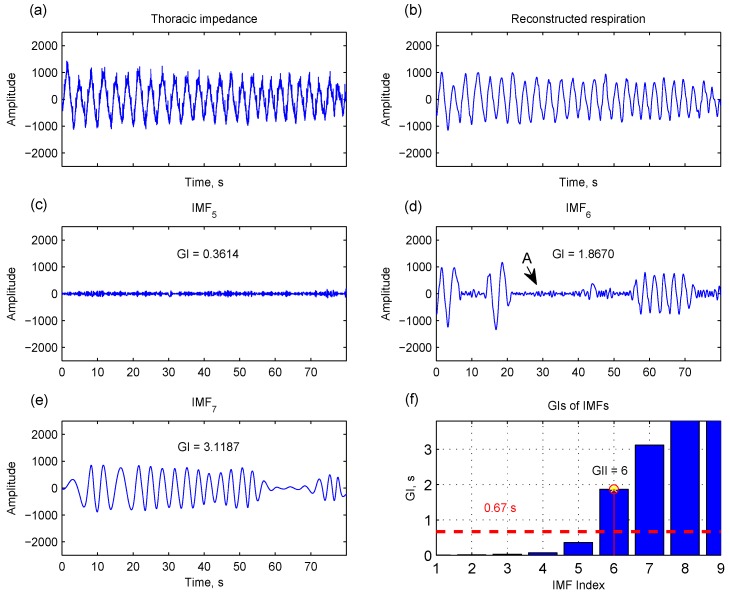
The global upper interval (GI), the mean of the 25% largest zero-crossing intervals, is computed for each intrinsic mode function (IMF). IMF_5_ has a smaller GI because its major content is noise (**c**). IMF_6_ and IMF_7_ contain a respiratory component, yielding a higher GI (**d**,**e**). Only considering upper intervals for computing GI is meant to avoid the effect of shorter zero-crossing intervals that are mainly attributed to noise (indicated by arrow A in IMF_6_). The IMFs with GI > 0.67 s (**f**) and a residual signal can be used to reconstruct a respiration signal (**b**).

In order to determine IRRI, two indexes are derived from each IMF. The first one is the global upper interval index (GII):

(6) $$\text{GII}=\min\{j|{\text{GI}}_{j}>0.67\text{ s}\}$$

where GI*_j_* is the mean of the 25% largest zero-crossing intervals in IMF*_j_*. As shown in Figure 6, IMF_5_ has a smaller GI because its major content is noise. IMF_6_ and IMF_7_ have a higher GI for containing respiratory components. Only including upper intervals for computing GI is meant to avoid the effect of shorter zero-crossing intervals that are attributed to noises (indicated by arrow A in Figure 6d). GII is the index that all GIs after this index (*j* ≥ GII) are greater than 0.67 s (Figure 6f). Selecting IMFs with GI > 0.67 s corresponds to considering oscillatory components with frequencies below 0.75 Hz (<45 breaths/min equivalently) that cover the normal respiratory rate and most of the abnormal respiratory rate [17,18].

**Figure 7:**
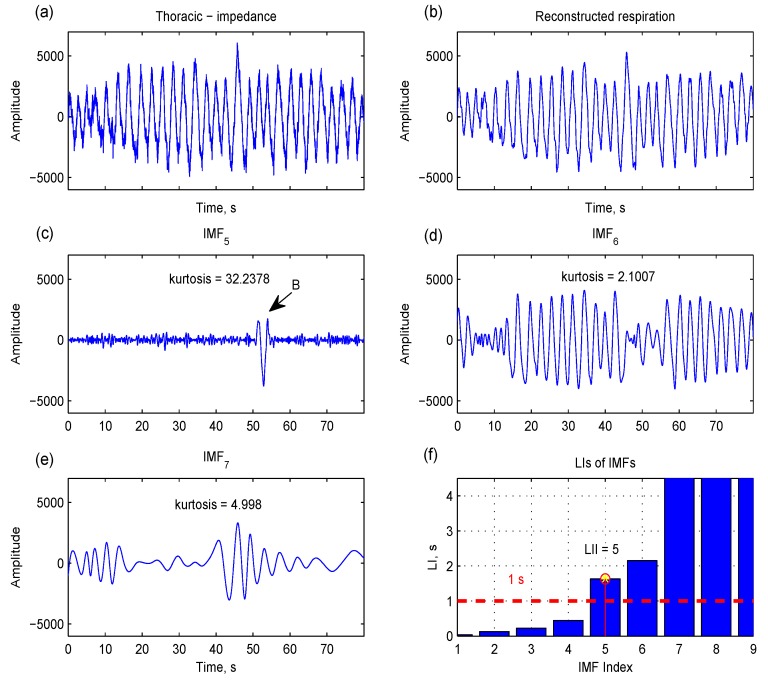
The largest interval (LI), the largest zero-crossing interval in intrinsic mode function (IMF), can catch the local oscillatory component whose frequency is lowest in each IMF (indicated by arrow B in IMF_5_). Since this local component is distinct compared to the rest of the components, IMF_5_ also has a high kurtosis (**c**). The IMFs with LI > 1 s (**f**) and a residual signal can be used to reconstruct a respiration signal (**b**).

The second index is the largest interval index (LII):

(7) $$\text{LII}=\min\{j|{\text{LI}}_{j}>1\text{ s}\}$$

where LI*_j_* is the largest zero-crossing interval in IMF*_j_*. The largest interval reflects a kind of local property. As shown in Figure 7c, there is a short respiratory component (indicated by arrow B) in IMF_5_, and this short oscillatory property can be captured by LI. LII is the index that all LIs after this index (*j* ≥ LII) are greater than 1 s (equivalent frequencies less than 0.5 Hz) (Figure 7f). If the short component is distinct from the rest of the components in the IMF *h*(*t*), this IMF has a high kurtosis:

(8) $$\text{kurt\{}h(t)\text{\}}=\frac{E\{{\left(h(t)-\mu \right)}^{4}\}}{\sigma^{4}}$$

where μ is the mean of *h*(*t*), σ is the standard deviation of *h*(*t*), and *E* is the expected value operation.

IMFs with GI > 0.67 s are available in most respiration reconstructions; that is, IRRI is set to GII in most cases. In some cases, there are short, distinct respiratory components (as shown in the IMF_5_ of Figure 7), yielding a high kurtosis. Therefore, the IRRI is set to LII in this situation:

(9) $$\text{IRRI}=\{\begin{array}{cc} \text{LII} & {\text{if kurt(IMF}}_{\text{LII}}\text{)}>\text{10}\\ \text{GII} & \text{otherwise} \end{array}$$

#### 2.2.4. Instantaneous Frequency and Amplitude

The Hilbert transform is a linear operator that takes a signal *h*(*t*), and produces another signal *g*(*t*):

(10) $$g\text{(}t\text{)}=\frac{1}{\pi }P\int_{-\infty }^{\infty }\frac{h(t^{\prime })}{t-t^{\prime }}dt^{\prime }$$

where *P* is the Cauchy principle value. The Hilbert transform is most often used to derive the analytic representation of the signal:

(11) u(t)=h(t)+jg(t)

The analytic signal is also expressed by instantaneous amplitude and phase:

(12) $$u(t)=a(t)\cdot e^{j\text{θ}(t)}$$

where $a(t)={\left[h^{2}(t)+g^{2}(t)\right]}^{1/2}$ and $\text{θ}(t)=\tan^{-1}\left(\frac{g(t)}{h(t)}\right)$.

A local restriction used to define a meaningful instantaneous frequency physically is that the signal should be symmetric with respect to the local zero mean. The IMF is defined to satisfy this local restrictive condition. Therefore, each IMF can have instantaneous frequency uniquely and meaningfully from an instantaneous phase:

(13) $$\omega (t)=\frac{d\text{θ}(t)}{dt}$$

### 2.3. Evaluation

For assessing the capability of the proposed method in estimating instantaneous respiratory frequency, 80 s thoracic impedance and 80 s oronasal airflow were extracted from the eleven 3 min physical activities from each dataset including supine, left-lateral lying, right-lateral lying, sitting, standing, slow walking, fast walking, slow running, fast running, standing in recovery, and supine in recovery. Most segments were selected from central portions and shifted forward or backward if covering motion disturbances. Both thoracic impedance and oronasal airflow were decomposed into several IMFs by EMD. For thoracic impedance, respiration-related IMFs were identified based on the IRRI rule. Since the oronasal airflow had a well-documented sinusoidal form, all decomposed IMFs were selected.

The respiratory amplitude was computed from all respiration-related IMFs as follows:

(14) $$A_{r}={\left(\sum_{j=IRRI}^{n}A_{j}^{2}\right)}^{1/2}$$

and the respiratory frequency was derived by aggregating the frequencies of all respiration-related IMFs:

(15) $$\omega_{r}=\sum_{j=IRRI}^{n}A_{j}^{2}\omega_{j}/\sum_{j=IRRI}^{n}A_{j}^{2}$$

where *A_j_* and ω*_j_* are the amplitudes and frequencies of IMF*_j_*. In order to reduce the variance of respiratory estimation, the derived instantaneous respiratory properties (Equations (14) and (15)) were divided into consecutive 1 s windows with 50% overlapping. In each window, the representative frequency was defined as the median value of all instantaneous frequencies and the representative amplitude was defined as the median value of all instantaneous amplitudes. The representative respiratory frequencies based on thoracic impedance were compared with those based on oronasal airflow using correlation analysis.

## 3. Results and Discussion

Table 1 and Table 2 list correlation coefficients between the respiratory frequencies estimated from thoracic impedance and those from oronasal airflow on the basis of different window sizes by the EMD-based method and the Fourier-based method, respectively. In the Fourier-based method, the thoracic impedance or oronasal airflow within the specified window was taken for the Fourier transform with zero padding to 20 s. The respiratory frequency was given by the frequency of the maximum spectral peak. Similarly, the correlation analysis was performed. The higher the correlation coefficient is, the more similarity between the respiratory frequencies derived from thoracic impedance and oronasal airflow. Although the mechanics of thoracic expansion and distraction may be different in various postures or dynamic physical activities, the EMD-based method provides higher cross correlations than the Fourier-based method when the specified window size is smaller than 4 s no matter whether it is a motion state (walking, running) or a static state (upright posture, supine, lateral lying).

**Table 1:** Correlation coefficients between the respiratory frequencies estimated from thoracic impedance and those from oronasal airflow using empirical mode decomposition.

Window Size, s	Motion State			Static State			
	Walking	Running	Both	Upright	Supine	LL	All
5	0.8071	0.8121	0.8205	0.7743	0.8048	0.7133	0.7648
4	0.7918	0.7958	0.8023	0.7546	0.7961	0.6820	0.7509
3	0.7672	0.7717	0.7761	0.7288	0.7803	0.6500	0.7296
2	0.7228	0.7280	0.7306	0.6891	0.7511	0.6047	0.6981
1	0.6511	0.6700	0.6602	0.6329	0.6963	0.5426	0.6485

The motion state includes walking (slow and fast) and running (slow and fast). The static state includes upright posture (sitting, standing, and standing in recovery), supine (at rest and in recovery), and lateral lying (LL: Left-lateral lying and right-lateral lying).

**Table 2:** Correlation coefficients between the respiratory frequencies estimated from thoracic impedance and those from oronasal airflow using Fourier transform.

Window Size, s	Motion State			Static State			
	Walking	Running	Both	Upright	Supine	LL	All
5	0.9255	0.9604	0.9367	0.8707	0.8366	0.7456	0.8636
4	0.8199	0.8763	0.8275	0.8023	0.7360	0.6483	0.8013
3	0.6395	0.7431	0.6708	0.6103	0.5427	0.4313	0.5782
2	0.3043	0.4609	0.4354	0.1822	0.0827	0.0465	0.1377
1	−0.0957	−0.1118	−0.1230	−0.0715	0.0213	−0.0188	−0.0384

The motion state includes walking (slow and fast) and running (slow and fast). The static state includes upright posture (sitting, standing, and standing in recovery), supine (at rest and in recovery), and lateral lying (LL: left-lateral lying and right-lateral lying).

Instantaneous respiratory estimation based on a longer window (e.g., 5 s) is beneficial to delineate steady-state breathing patterns. In contrast, analysis using a shorter window (e.g., 1 s) can catch the changes of respiratory features and help detecting the onset and the offset of respiratory episode.

The correlation coefficient based on the EMD-based method is about 0.64–0.82 depending on the window size. The possible reason is that the thoracic impedance-based respiration and the oronasal airflow-based respiration have different rationales in measurement, and both measurements are contaminated by different type of disturbances. The thoracic impedance is usually disturbed by thoracic motions and the oronasal airflow is affected by mouth-thorax activities. These disturbances will affect the estimation of respiratory frequencies, in particular the estimation based on a short window size.

Each respiratory disorder has its own respiratory pattern. Obstructive sleep apnea is caused by airway narrowing and collapse in the throat. Repetitive stopping or slowing of breathing is the major symptom. Although the airflow is blocked during airway obstruction, small respiratory effort is still observed in the thorax. Central sleep apnea, including Cheyne–Stokes respiration, is frequently observed in patients with chronic heart failure. It presents another respiratory pattern: progressively deeper breathing (hyperpnoea), followed by a gradual decrease (hypopnea) and a temporary stop (apnea) of breathing. The respiratory amplitude as well as frequency are different from normal breathing and change during the episode. The EMD will be beneficial to catch the time-related changes of respiratory characteristics.

**Figure 8:**
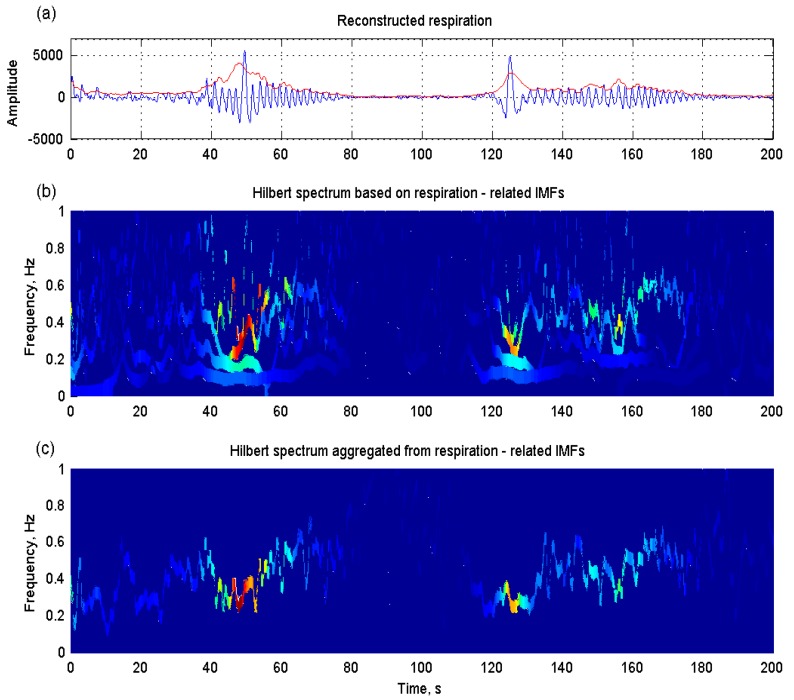
(**a**) Cheyne–Stokes respiration and its instantaneous amplitude (red line) reconstructed from the thoracic impedance by the proposed EMD-based method in a patient with congestive heart failure; (**b**) detailed respiratory properties displayed in the Hilbert spectrum estimated from respiration-related intrinsic mode functions (IMFs) individually; (**c**) a clear time-related respiratory pattern displayed in the aggregated spectrum from all respiration-related IMFs.

Figure 8 illustrates an example of shortness of breath, also named Cheyne–Stokes respiration, in a patient with congestive heart failure. Figure 8a shows the reconstructed respiration from the thoracic impedance by the proposed EMD-based method. Its instantaneous amplitude (red line) was computed based on Equation (14). The Hilbert spectrum derived from respiration-related IMFs individually displays detailed respiratory properties (Figure 8b), whereas the aggregated spectrum from all respiration-related IMFs shows a clear time-related respiratory pattern (Figure 8c).

Long-term ambulatory Holter electrocardiogram recordings and analysis are widely used to detect cardiac arrhythmia or probe autonomic nervous function through heart rate variability analysis. Polysomnography recording helps detect respiratory disorders during sleep. The purposes of these two examinations are different and are usually performed individually. However, many patients with cardiovascular dysfunction also have respiratory disorders. The occurrence of central sleep apnea has been demonstrated to be relative to clinical outcomes, atrial arrhythmia, or ventricular arrhythmia in patients with heart failure [2,3,19]. Moreover, the autonomic nervous system and respiratory control system have a reciprocal interaction. Reduced cardio-respiratory coupling was observed in severe obstructive sleep apnea compared to patients with no or mild obstructive sleep apnea [20]. Incompletely developed cardio-respiratory coupling was noted in very pre-term neonates [21].

Impedance plethysmography needs little adjustment to maintain close attachment between a sensor and the human body since its sensor is just the human body itself. Compared to polysomnography monitoring, impedance plethysmography with other physiological signals such as electrocardiography, *etc.* is more convenient and comfortable, and is quite suitable for the assessments of respiratory and cardio-respiratory disorders in patients with sleep apnea or cardiovascular impairment, preterm infants, and so on. Similar to respiratory sensor belts, thoracic impedance is also disturbed by upper-body movements. Because the interference of motion disturbance upon the measured signal is not linear, the induced motion artifacts cannot be easily removed by linear filtering. The proposed motion artifact detection can isolate the affected portion to avoid incorrectly interpreting it as a respiratory component, whereas this is important for analyzing long-term respiration.

## 4. Conclusions

The impedance plethysmography provides a convenient, comfortable measurement for ambulatory respiratory monitoring. The proposed artifact detection and replacing algorithm avoids misinterpreting motion artifacts as respiratory components and interfering with EMD processing. A novel rule based on both the global and local properties of IMFs can efficiently identify respiration-related IMFs where the derived respiratory property based on small window size is well delineated, whereas it fails by the Fourier transform-based method.
